# Altered morphological cortical thickness and disrupted network attributes and its relationships with drug use characteristics and impulsivity in abstinent male subjects with methamphetamine use disorder

**DOI:** 10.1017/S0033291725101165

**Published:** 2025-07-25

**Authors:** Dan Luo, Danlin Shen, Huiting Luo, Jiaxi Zhang, Qiao Tang, Mingfeng Lai, Jia-jun Xu, Jing Li

**Affiliations:** Mental Health Center, West China Hospital of Sichuan University, Chengdu, China

**Keywords:** cortical thickness, graph theory, methamphetamine use disorder, structure covariance network, trait impulsivity

## Abstract

**Background:**

Methamphetamine (METH) dependence is a globally significant public health concern with no efficacious treatment. Trait impulsivity is associated with the initiation, maintenance, and recurrence of substance abuse. However, the presence of these associations in METH addiction, as well as the underlying neurobiological mechanisms, remains incompletely understood.

**Methods:**

We scanned 110 individuals with METH use disorder (MUDs) and 55 matched healthy controls (HCs) using T1-weighted imaging and assessed their drug use characteristics and trait impulsivity. Surface-based morphometry and graph theoretical analysis were used to investigate group differences in brain morphometry and network attributes. Partial correlations were conducted to investigate the relationships between brain morphometric changes, drug use parameters, and trait impulsivity. Mediation analyses examined how trait impulsivity and drug craving influenced the link between brain morphometric change and MUD severity in patients.

**Results:**

MUDs exhibited thinner thickness in the left fusiform gyrus and right pars opercularis, as well as diminished small-world properties in their structural covariance networks (SCNs) compared to HCs. Furthermore, reduced cortical thickness in the right pars opercularis was linked to motor impulsivity (MI) and MUD severity, and the association between the right pars opercularis thickness and MUD severity was significantly mediated by both MI and cue-induced craving.

**Conclusions:**

These findings suggest that MUDs exhibit distinct brain structural abnormalities in both the cortical thickness and SCNs and highlight the critical role of impulse control in METH addiction. This insight may offer a potential neurobiological target for developing therapeutic interventions to treat addiction and prevent relapse.

## Introduction

Methamphetamine (METH) is a globally pervasive substance of abuse, with a particularly pronounced prevalence in China where it constitutes a substantial illicit drug issue (United Nations Office on Drugs and Crime, 2023). Chronic METH use induces profound neurotoxicity (Jayanthi, Daiwile, & Cadet, 2021), manifesting in structural brain alterations, cognitive deficits, and heightened impulsivity, a core trait linked to addiction initiation, maintenance, and relapse (Verdejo-Garcia & Albein-Urios, 2021). Despite advances in neuroimaging, the neurobiological mechanisms underlying METH use disorder (MUD) remain poorly characterized, impeding the development of targeted therapies (London, 2016; Ruan et al., 2020). A critical gap lies in the limited integration of advanced morphometric techniques and network-level analyses to unravel how structural reorganization mediates behavioral pathologies in MUD.

Prior neuroimaging studies predominantly employed voxel-based morphometry (VBM) to identify volumetric changes in temporal (Nakama et al., 2011; Thompson et al., 2004), frontal (MacDuffie et al., 2018; Schwartz et al., 2010), and subcortical regions among METH users (Jan et al., 2012). However, VBM conflates cortical thickness (CT) and surface area, which are distinct neurobiological processes governed by separate genetic and developmental pathways (Winkler et al., 2010). Surface-based morphometry (SBM) addresses this limitation by separately quantifying CT (reflecting dendritic arborization and synaptic density) and surface area (linked to cortical column organization) (Hu et al., 2022). Importantly, CT abnormalities may better capture METH-induced synaptic toxicity (Panizzon et al., 2009; Rakic, 1988; Schaer et al., 2008), as animal models demonstrate dendritic spine loss in prefrontal regions following chronic exposure (Nagy et al., 2024). Despite its potential, SBM remains underutilized in MUD research, and none investigating structural covariance networks (SCNs), a powerful framework to map coordinated variations in gray matter across brain regions (Mechelli, Friston, Frackowiak, & Price, 2005).

SCNs reflect synchronized variations in morphometric features across anatomically or functionally connected regions, offering insights into large-scale brain organization (Ottino-González & Garavan, 2022). Emerging evidence suggests that psychostimulants like cannabis disrupt SCN topology, particularly in frontostriatal circuits governing inhibitory control (Li & Xu, 2024). However, analogous studies in METH addiction are conspicuously absent. This gap is striking given recent functional MRI findings showing aberrant local efficiency in METH users’ default-mode and salience networks (Mansoory, Allahverdy, Behboudi, & Khodamoradi, 2022). Since structural networks scaffold functional connectivity, SCN analyses could reveal enduring neuroplastic adaptations that perpetuate addiction cycles: a hypothesis yet to be tested.

Impulsivity, a transdiagnostic feature of addiction (Verdejo-Garcia & Albein-Urios, 2021), is exacerbated by METH-induced prefrontal-striatal dysregulation (Jahanshahi, Obeso, Rothwell, & Obeso, 2015). Cross-sectional studies associate impulsivity with reduced cortical morphometry in cocaine users (Kaag et al., 2014), but directional relationships remain unclear. Crucially, no study has examined whether CT abnormalities mediate the link between METH exposure and impulsivity, nor how these effects propagate through SCNs. Addressing this could elucidate whether impulsivity arises from neurodevelopmental vulnerabilities or neurotoxic sequelae, a distinction that holds significant therapeutic implications.

Based on this background, this study integrates SBM and graph theory to investigate morphometric specificity in individuals with MUD (MUDs) whether exhibiting CT reductions in circuits critical for impulse control, and network dysregulation in SCN topological properties. In addition, the mediating role of impulsivity and craving for METH in the relationship between abnormal brain morphometry and addiction severity was also examined. Our findings offer a structural basis for functional network anomalies observed in METH users and provide insights for interventions aimed at enhancing circuit-specific plasticity to mitigate relapse risk.

## Materials and methods

### Participants

A total of 110 male participants were recruited from the Chengdu Compulsory Detoxification Center between January 2018 and October 2019. These individuals fulfilled following inclusion criteria: they were aged 18–55 years, identified Han Chinese, were right-handed, and met the diagnostic criteria for MUD as specified in the Chinese version of the Structured Clinical Interview for Diagnostic and Statistical Manual of Mental Disorders, fifth edition (DSM-V), as determined by a board-certified psychiatrist. The HCs (*N* = 55) were recruited from the local community through advertisements and were matched with the MUD group in terms of gender, age, and ethnicity.

The exclusion criteria encompassed the following conditions: the presence of a substance use disorder other than METH or nicotine as confirmed by urine toxicology upon participants’ admission to the facility; a history of mental disorders unrelated to substance use as assessed by the SCID-5 and clinical records, such as bipolar disorder and attention deficit hyperactivity disorder (ADHD); the presence of specific medical conditions, including cardiovascular disease, severe liver disease, or kidney disease; a history of brain injury or loss of consciousness exceeding 10 minutes; left-handedness; and contraindications for undergoing magnetic resonance imaging (MRI) scanning. A total of 18 potential participants were excluded: 10 due to polysubstance use (e.g. opioids/cocaine-positive urine tests), 4 for major psychiatric comorbidities (e.g. bipolar disorder), and 4 for incomplete data. Finally, the study included a total of 155 male participants.

This study was approved by the West China Hospital of Sichuan University Biomedical Research Ethics Committee, and it adhered to the tenets of the Declaration of Helsinki. All the participants who voluntarily took part in this study were informed in advance about its purpose, procedures, and the possible risks and benefits. They were also assured that all information collected would remain anonymous and confidential. Participation was entirely voluntary, and refusal to participate would not affect their current or future treatment in any way. Those who agreed to participate signed an informed consent form. To minimize coercion risks, data collection was conducted by independent researchers who were not affiliated with the facility staff. Participants were clearly informed that their responses would have no impact on treatment outcomes or access to services.

### Clinical and impulsivity data acquisition

#### General questionnaire

All participants underwent a structured interview conducted by a certificated psychiatrist. The interview collected data on variables, including age, ethnicity, years of education, smoking status, drinking status, combined use of other substances, initial age of METH use, METH use duration, METH dose taken per time, METH use frequency, and duration of METH abstinence. The history of METH use was corroborated through urine toxicology screening, which was administered immediately upon the participants’ entry into the facilities.

#### Visual analogue scale

The visual analogue scale (VAS) is extensively employed to assess the current intensity of psychological craving for various substances. In accordance with the methods reported in prior literature (Shen et al., 2016), we developed a presentation comprising 34 METH-related pictures, such as drug use environments, paraphernalia, drug powder, and drug use scenarios. Each set of 34 images was displayed for a duration of 5 minutes and presented twice. Following exposure to the images, participants were instructed to select a number from 0 (no craving) to 10 (strongest imaginable craving) that best represented their current level of craving for the drug, with higher numbers indicating a more intense psychological craving for METH.

#### Barratt Impulsiveness Scale, version 11

Impulsivity was assessed using the Chinese version of the Barratt Impulsiveness Scale, version 11 (BIS-11), which is the most widely utilized questionnaire for measuring impulsiveness (An, Phillips, & Conner, 2010; Yao et al., 2007). The BIS-11 comprises 30 items designed to evaluate three dimensions of impulsivity: attentional impulsivity (AI), characterized by cognitive deficits or difficulty in task completion; motor impulsivity (MI), defined as acting without considering consequences; and nonplanning impulsivity (NPI), which pertains to a lack of future planning and a tendency toward an irregular lifestyle. Each item on the BIS-11 is rated on a scale ranging from 1 (never) to 5 (always). Higher scores indicate higher levels of impulsivity (Patton, Stanford, & Barratt, 1995).

#### MUD severity

The severity of MUD was assessed using the latest version of DSM-5, which provides criteria for substance use disorders. The DSM-5 offers a streamlined diagnostic framework that emphasizes severity rather than distinctions between abuse and dependence. It categorizes substance use disorders based on the number of symptoms present: No problem (0–1 symptoms), Mild (2–3 symptoms), Moderate (4–5 symptoms), and Severe (6 or more symptoms).

### MRI data acquisition and preprocessing

All participants were scanned on a 3.0 T MR scanner (Achieva; Philips, Amsterdam, the Netherlands) using an eight-channel phased-array coil in the department of radiology. High-resolution T1-weighted images were obtained by a 3D magnetization-prepared rapid gradient-echo (MPRAGE) sequence. The parameters for image acquisition were as follows: TR = 8.37 ms, TE = 3.88 ms, flip angle = 7°, in-plane matrix resolution = 256 × 256, FOV = 240 mm × 240 mm, and number of slices = 188. The quality of the brain images was examined immediately after each scan, and scans were repeated if gross distortions were found.

The preprocessing of structural T1-weighted images and the reconstruction of brain surface were performed using the FreeSurfer package version 7.2 (http://surfer.nmr.mgh.harvard.edu) through the ‘recon-all’ pipeline. Specifically, the automatic process encompassed several steps: motion correction, skull stripping via a deformable template model, automated registration to Talairach space, segmentation of subcortical white matter and deep gray matter volumetric structures, intensity normalization, tessellation of GM and WM boundaries, automated topology correction and surface deformation following intensity gradients, surface inflation and registration to a spherical atlas, and cortical parcellation (Desikan et al., 2006; Fischl et al., 2004). The default parcellation scheme provided by FreeSurfer, known as the Desikan-Killiany atlas, contains 68 cortical and subcortical regions and has been widely used in the previous studies (Desikan et al., 2006). CT was operationalized as the mean shortest distance between the white matter/gray matter (WM/GM) boundary and GM/cerebrospinal fluid boundary at each point (Fischl & Dale, 2000). For quality control purposes, the entire cortex of each participant underwent visual inspection, and any segmentation inaccuracies were manually corrected (Bruce Fischl et al., 2002).

### Network construction

**Figure 1.**
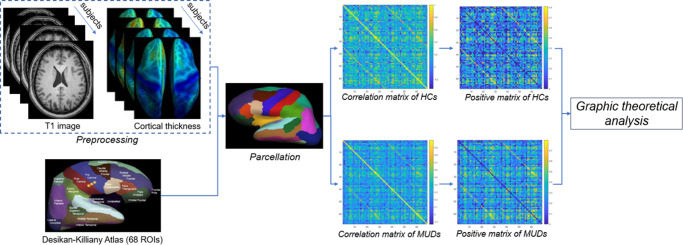
Schematic diagram of steps involved in producing and evaluating structural covariance networks based on the cortical thickness of patients with methamphetamine and healthy controls.

We used the Desikan–Killiany atlas (Desikan et al., 2006) for SBM to parcellate the whole-brain CT into 68 regions of interest (ROIs), which served as the network nodes. SCN construction was performed using the Brain Connectivity Toolbox (http://www.brain-connectivitytoolbox.net/) (Bullmore & Bassett, 2011) on the Matlab2020b platform. Age, educational level, current smoking status, ever drinking status, and the estimated total intracranial volume (eTIV) were included as covariates. Partial correlation analyses were performed to calculate the structural covariance between each pair of corrected ROIs in both the MUD group and HC group. Initially, we generated the structural correlation matrix, which was subsequently transformed into positive values to facilitate the calculation of topological properties. The range of density was from 0.05 to 0.4 with an interval of 0.01. Subsequently the graph theoretical analysis was applied to quantify the topological properties (Figure 1).

### Statistical analyses

Independent samples t tests or nonparametric tests were used to compare variables among groups for normally or nonnormally distributed data, respectively. Categorical variables were analyzed using the chi-square test. Comparisons of CTs across 68 ROIs between groups were conducted, incorporating age, educational level, current smoking status, ever drinking status, and eTIV as covariates.

Graph theoretical analyses of group differences in the topological properties of SCNs primarily employed permutation test theory. For both the HCs and MUDs, between-group differences in graph theory metrics (i.e. Sigma, Lambda, Gamma, Cp, E_global_, E_local_, Lp) were evaluated using two-sided permutation tests at each density level. Nonparametric permutation testing was required as metrics were computed at the group level, yielding a single value per group. To mitigate the dependence of results on a single threshold, area under the curve (AUC) analyses were conducted. Individuals were randomly shuffled among groups 1000 times, and two-sided AUC tests were conducted. The observed AUC differences were then compared to critical values derived from the 95th percentile confidence intervals of the distribution of permuted AUC differences.

Partial correlation analyses were conducted to assess the relationships among CT of brain regions exhibiting intergroup differences, drug use parameters, and trait impulsivity. In addition, mediation analyses were performed to explore the mediating roles of trait impulsivity and drug craving in the relationship between CT of specific brain region and addiction severity in MUDs, adjusted for age, educational level, current smoking status, history of alcohol consumption, total intracranial volume, and abstinent duration of METH. General statistical analyses were performed using SPSS software (version 26.0), while the mediation model was estimated with 5000 bootstraps using the PROCESS macro (version 3.2) for SPSS (Hayes & Preacher, 2014; Preacher & Hayes, 2008). *p* < 0.05 was defined as statistically significant, and Bonferroni correction was used to address the issue of multiple comparisons.

## Results

### Demographic data, drug use, and trait impulsivity

A total of 155 male participants were included in this study, comprising 52 healthy controls (HCs) and 103 individuals diagnosed with MUD according to DSM-V criteria. Their demographic information is presented in Table 1. There was no statistical difference in age between the two groups (*p* > 0.05). However, significant differences were observed in educational level (*p* < 0.001), current smoking status (*p* < 0.001), and ever drinking status (*p* < 0.001). In addition, Table 1 provides relevant characteristics of METH use among MUDs, who reported significantly higher scores in MI, AI, NPI, and BIS-11 score compared to HCs (*p* < 0.001).

**Table 1.** Demographic information of healthy controls and patients with methamphetamine use disorder

Variables	HC (*N* = 52)	MUD (*N* = 103)	t/χ^2^	*p* value
Age, years	29.48 ± 5.72	29.15 ± 8.19	0.296	0.792
Educational level			54.395	**<0.001**
≤9 years	1 (1.9%)	66 (64.1%)		
>9 years	51 (98.1%)	37 (35.9%)		
Current smoking			68.589	**<0.001**
No	31 (59.6%)	2 (1.9%)		
Yes	21 (40.4%)	101 (98.1%)		
Ever drinking			16.400	**<0.001**
No	41 (78.8%)	46 (44.7%)		
Yes	11 (21.2%)	57 (55.3%)		
MA abstinence duration, months	NA	8.99 ± 5.52	–	NA
Initial age of substance use, years	NA	23.11 ± 8.03	–	NA
MA use duration, months	NA	51.87 ± 39.74	–	NA
MA average dose per use, g	NA	0.56 ± 1.03	–	NA
MA use frequency			–	NA
At least once per day	NA	16 (15.5%)		
Every 2–3 days	NA	25 (24.3%)		
At least once a week	NA	28 (27.2%)		
Twice to three times a month	NA	12 (11.7%)		
Once a month	NA	3 (2.9%)		
Less than once a month	NA	19 (18.4%)		
VAS score	NA	1.32 ± 1.96	–	NA
MUD severity			–	NA
Mild	NA	13 (12.6%)		
Moderate	NA	37 (35.9%)		
Severe	NA	53 (51.5%)		
BIS–11 score	64.63 ± 14.09	82.12 ± 17.85	−6.155	**<0.001**
Motor impulsivity	21.13 ± 5.28	25.04 ± 7.34	−3.792	**<0.001**
Attentional impulsivity	21.73 ± 4.89	27.768 ± 7.58	−5.897	**<0.001**
Nonplanning impulsivity	21.77 ± 6.10	29.55 ± 8.63	−6.488	**<0.001**

*Note:* Continuous data are presented as the mean ± standard deviation, and categorical data are presented as count (percentage).

Abbreviations: HC, healthy control group; MUD, methamphetamine use disorder group; MA, methamphetamine; VAS, visual analogue scale; BIS-11, Barratt Impulsiveness Scale, version 11.

### Group differences in brain CT and topological metrics of SCNs

**Figure 2.**
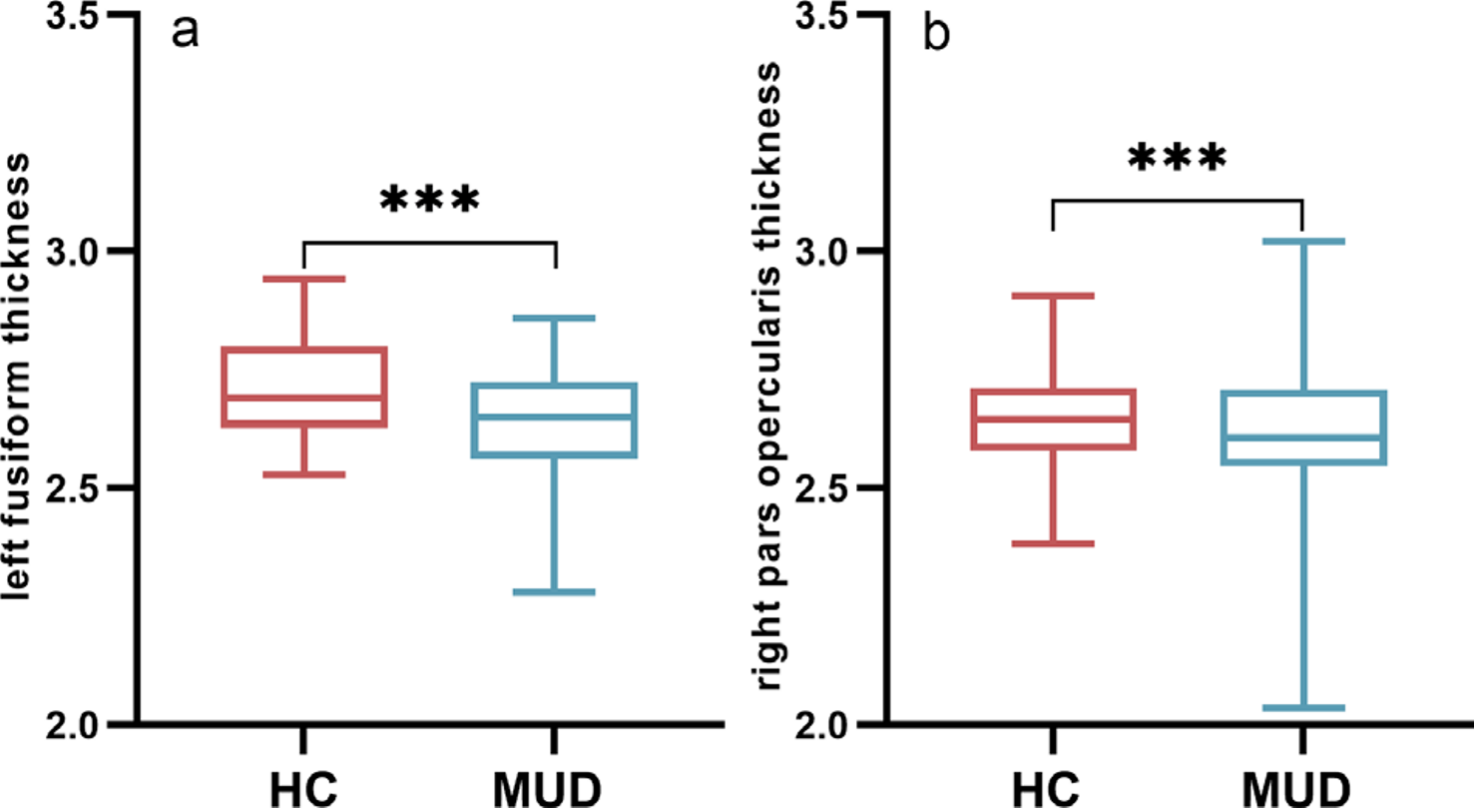
Differences in brain cortical thickness between healthy controls and individuals with methamphetamine use disorder. (a) Group difference in left fusiform thickness; (b) group difference in right pars opercularis thickness. *Note:* CT, ‘cortical thickness’; HC, ‘healthy control group’; MUD, ‘methamphetamine use disorder group’. ***: *p* < 0.001, Bonferroni corrected.

Age, educational attainment, current smoking status, history of alcohol consumption, and eTIV were incorporated as covariates in the comparative group analyses. As shown Figure 1, compared to HCs, MUDs exhibited significantly reduced CT in the left fusiform (*p* < 0.001, Bonferroni corrected, Figure 2a) and right pars opercularis (*p* < 0.001, Bonferroni corrected, Figure 2b).

**Figure 3.**
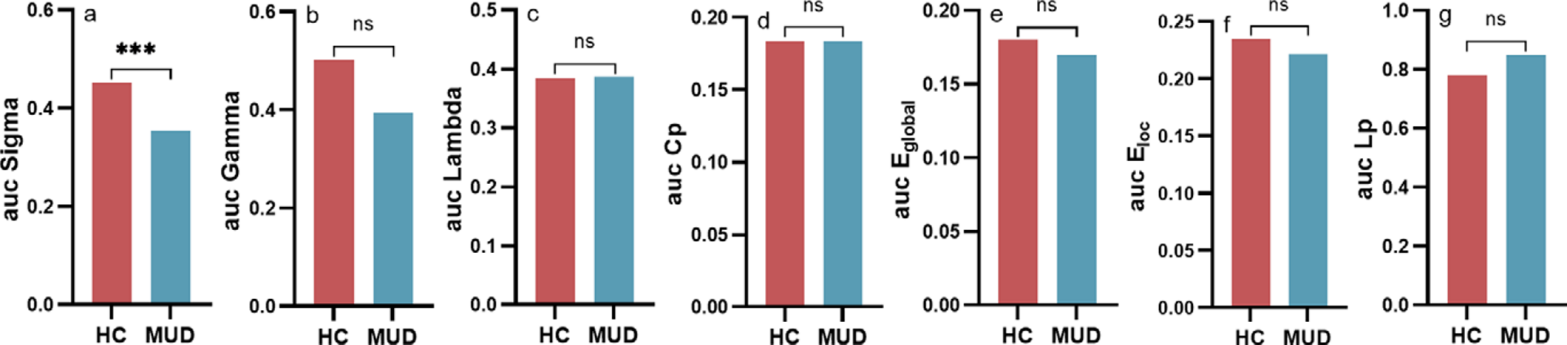
Differences in group level brain structure covariance network between healthy controls and individuals with methamphetamine use disorder. (a–f) Group differences in Sigma, Gamma, Lambda, Cp, E_global_, E_loc_, Lp, respectively. *Note:* auc, ‘area under the curve’; Cp, ‘clustering coefficient’; E_global_, ‘global efficiency’; E_loc_, ‘local efficiency’; Lp, ‘the characteristic path length’; HC, ‘healthy control group’; MUD, ‘methamphetamine use disorder group’. ***: *p* < 0.001, Bonferroni corrected; ns, not significant.

We performed graph theoretical analyses across a spectrum of densities to explore differences in the group-level SCNs based on CT and found that MUDs showed significantly lower small-world attribution (*p* < 0.001, Figure 3a) in comparison to HCs. However, no significant differences were detected in the other global-level topological characteristics (*p* > 0.05, Figure 3b-g) and the nodal topological characteristics.

### Associations between the CT of specific brain regions, METH use parameters, and trait impulsivity in MUDs

**Figure 4.**
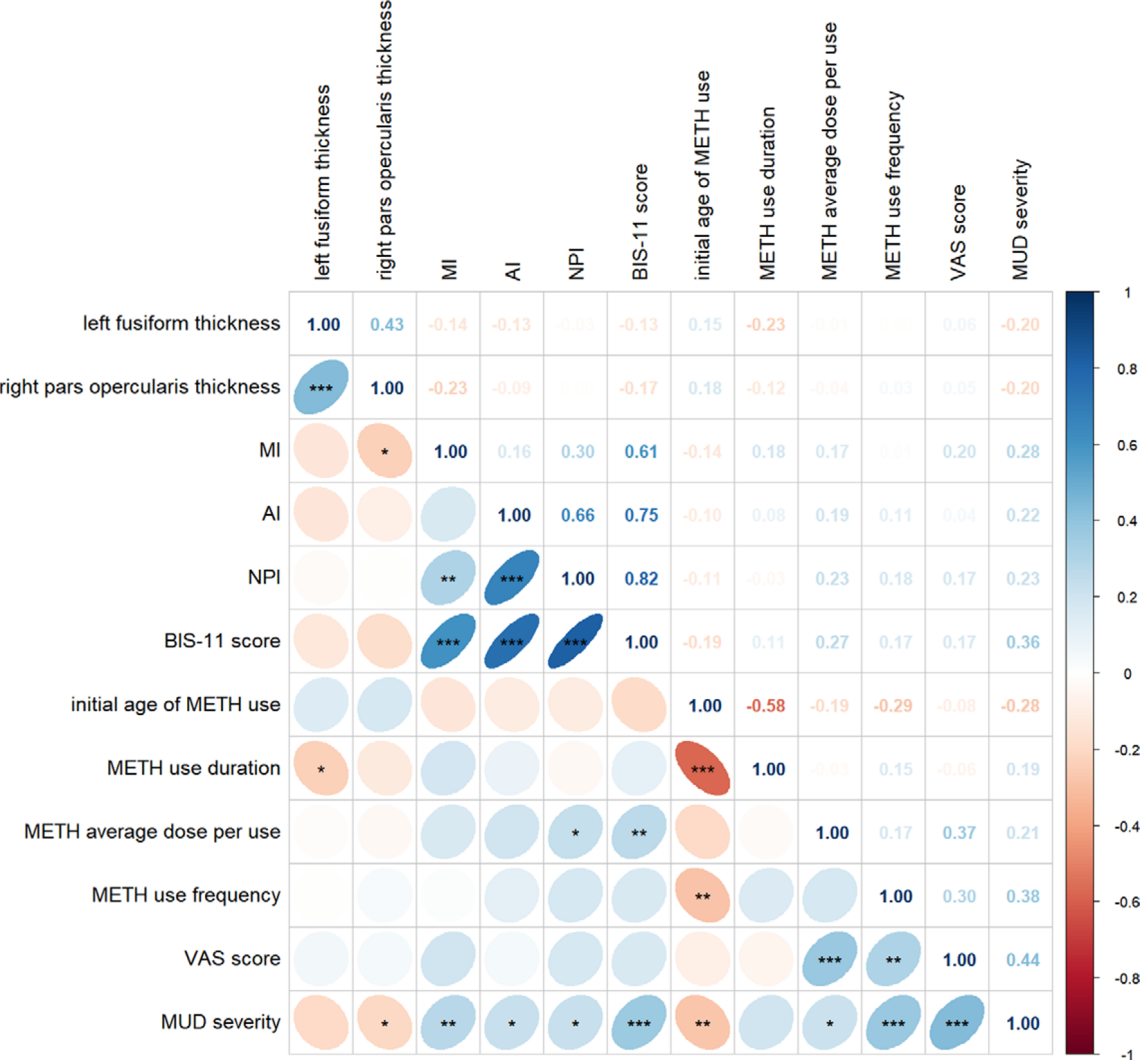
Results of partial correlation analysis among cortical thickness of specific brain regions, trait impulsivity, and drug use characteristics. *Note:* MI, ‘motor impulsivity’; AI, ‘attentional impulsivity’; NPI, ‘nonplanning impulsivity’; BIS-11, ‘Barratt Impulsiveness Scale, version 11’; MA, ‘methamphetamine’; VAS, ‘visual analogue scale’; MUD, ‘methamphetamine use disorder’. *: *p* < 0.05, **: *p* < 0.01, ***: *p* < 0.001.

Partial correlation analyses were conducted to investigate the potential associations between the cortical thickness of specific brain regions, characteristics of METH use, and trait impulsivity in MUDs. After controlling for age, educational level, current smoking status, history of alcohol consumption, abstinent duration of METH, and eTIV, it was observed that left fusiform thickness was negatively correlated with the duration of METH use (r = −0.232, *p* < 0.05, Figure 4), and right pars opercularis thickness was found to be negative association with MI (r = −0.233, *p* < 0.05, Figure 4) and the severity of METH addiction (r = −0.204, *p* < 0.05, Figure 4).

### Mediating role of trait impulsivity and drug craving between the right pars opercularis thickness and addiction severity

**Figure 5.**
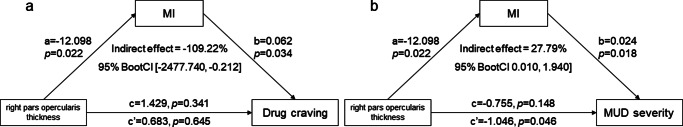
Mediating effects of motor impulsivity in the relationship among right pars opercularis thickness, drug craving, and addiction severity. (a) Mediating role of motor impulsivity between right pars opercularis thickness and drug craving; (b) Mediating role of motor impulsivity between right pars opercularis thickness and methamphetamine use disorder severity. *Note:* MI, ‘motor impulsivity’; MUD, ‘methamphetamine use disorder’.

**Figure 6.**
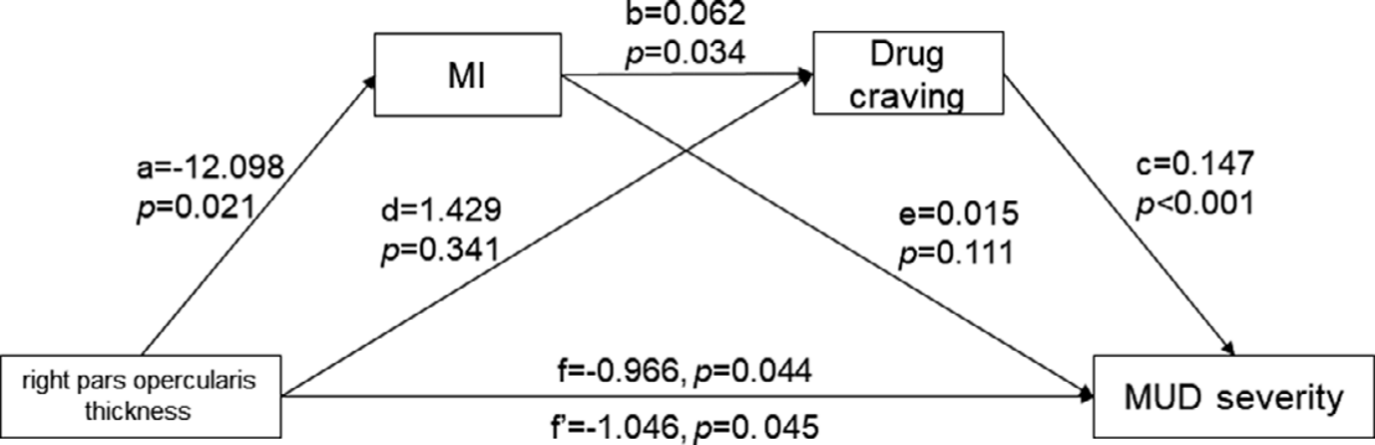
Association of right pars opercularis thickness (X) and methamphetamine use disorder severity (Y) mediated by motor impulsivity (M1) and drug craving (M2). *Note:* MI, ‘motor impulsivity’; MUD, ‘methamphetamine use disorder’.

Mediation analyses were conducted to examine the potential mediating role of trait impulsivity in the relationships among drug caving, right pars opercularis thickness, and addiction severity. After controlling for age, educational level, current smoking status, history of alcohol consumption, abstinent duration of METH, and eTIV, the results of simple mediation analyses revealed that MI significantly mediated the relation between right pars opercularis thickness and cue-induced craving for METH (indirect effect = −109.22%, 95%CI: [−2477.740, −0.212], Figure 5a), as well as the relation between right pars opercularis thickness and MUD severity (indirect effect = 27.79%, 95%CI: [0.010, 1.940], Figure 5b). Furthermore, additional chain mediation analyses were conducted for these variables. As illustrated Figure 6 and Table 2, the relationship between the thickness of the right pars opercularis and the severity of METH addiction was significantly mediated by MI and cue-induced craving (indirect effect = 10.52%, 95%CI: [0.009, 0.759]).

**Table 2.** The mediating effects of motor impulsivity and drug craving on the association between right pars opercularis thickness and addiction severity

Model pathways	β	SE	95% CI	
			Lower	Upper
Total effect	−1.046*	0.516	**−2.070**	**−0.021**
Direct effect	0.966*	0.473	**−1.906**	**−0.026**
Indirect effect via drug craving	0.211	0.245	−0.278	0.700
Indirect effect via motor impulsivity	−0.181	0.174	−0.674	0.047
Indirect effect via motor impulsivity and drug craving	−0.110*	0.073	**−0.331**	**−0.016**

Abbreviations: CI, confidence interval; SE, standard error.

* *p* < 0.05.

## Discussion

In this study, we investigated examined alterations in CT and SCNs in MUDs relative to age- and gender-matched HCs. Our findings revealed that MUDs exhibit thinner thickness in the left fusiform gyrus and right pars opercularis, as well as diminished small-world properties in their SCN s compared to HCs. In addition, we observed a correlation between the thickness of the right pars opercularis and MI and addiction severity. The association between the right pars opercularis thickness and the severity of METH addiction was significantly mediated by both MI and cue-induced craving. These findings suggest that MUDs exhibit distinct structural abnormalities in CT, with the right pars opercularius thickness serving as a neurobiological underpinning in the relationships among MI, drug craving, and addiction severity in this population.

### CTs differences between MUDs and HCs

Our observation of reduced CT in the left fusiform and right pars opercularis in MUDs compared to HCs adds nuanced insights to the conflicting literature on METH-induced cortical alterations. While previous studies reported greater CT in parietal (Yang et al., 2021) and frontal regions (Nie et al., 2020) or diffuse frontal parietal temporal thinning relative to controls (Petzold et al., 2022) in abstinent MUDs. There are three potential reasons likely explaining these discrepancies. First, our focused analysis on frontal-striatal circuits using ROI-based whole brain approaches contrasts with voxel-based whole brain approaches in prior research (Nie et al., 2020; Petzold et al., 2022; Yang et al., 2021) that may dilute signals from functionally critical subregions. Second, unlike studies with mixed abstinent durations, our cohort (mean 9 months abstinence) may capture enduring neurotoxicity rather than acute withdrawal effects. Third, there exists substantial heterogeneity among the recruited populations in the individual studies, including variables, such as polysubstance abuse, comorbidity with mental disorders, and varying sample sizes. A notable advantage of our study is its focus on a single substance of abuse and mental disorders excluded. Notably, the fusiform gyrus and right pars opercularis belong to distinct functional networks, the former to the ventral visual stream involved in drug cue processing (Wittemann et al., 2021), and latter to the fronto-basal ganglia hyperdirect pathway governing response inhibition (Aron, Robbins, & Poldrack, 2014), indicating that chronic METH may preferentially target circuits interfacing sensory salience and impulse control. In addition, the right pars opercularis as a cortical input node to the subthalamic nucleus positions it as a structural bottleneck for inhibitory control (Aron et al., 2014; Jahanshahi et al., 2015). Our finding aligns with addiction models positing that fronto-subthalamic disconnection underlies compulsive drug seeking (Brown, Upton, Craig, & Froeliger, 2023; Mirabella, 2021) but extends them by demonstrating structural (not just functional) degradation in this pathway.

### Small-world disruption: a network-level hallmark of MUD

The diminished small-world properties in MUDs’ structural covariance networks (SCNs) reveal a fundamental reorganization of brain architecture. Small-world topology balances local specialization (high clustering) and global integration (short path length), a configuration optimized for cognitive flexibility (Bullmore & Sporns, 2009). Our data suggest that METH addiction induces a shift in SCNs toward a more randomized state, mirroring findings in the functional networks of METH users (Luo et al., 2024). This phenomenon might reflect that excessive oxidative stress resulting from chronic METH exposure could lead to the degradation of hub regions, thereby disrupting their integrative capacity. The observed randomization might represent failed attempts to reroute information flow following damage to critical nodes (Tijms et al., 2013). Notably, this is the first demonstration of SCN randomization in MUDs. Unlike functional connectivity studies that capture dynamic states, SCNs reflect enduring structural scaffolding. Our findings bridge the gap between microscale synaptic loss and macroscale network dysfunction, offering a multilevel framework for addiction pathology.

### Associations among CT of specific brain regions, drug use parameters, and trait impulsivity

The left fusiform thickness was found to be negatively correlated with the duration of METH use, indicating that prolonged METH consumption accompanies processes leading to cortical thinning. This phenomenon can be attributed to the oxidative stress, excitotoxicity, inflammation, and neuroadaptations induced by METH, as documented in previous studies (Berman et al., 2008; Krasnova & Cadet, 2009; Paulus & Stewart, 2020). It is plausible that neuronal loss contributes to this cortical thinning (Berman et al., 2008). The fusiform gyri are traditionally implicated in the recognition of objects, faces, and bodies (Kim, Lee, Erlendsdottir, & McCarthy, 2014). In the context of MUDs, morphological alterations in the fusiform gyri may be more specifically linked to impairments in spatial working memory tasks, as indicated by recent findings (Wittemann et al., 2021), suggesting a significant association between this region and the pathophysiology of MUD. In addition, our study observed a negative correlation between the right pars opercularis thickness and both MI and addiction severity rather than AI and NPI. This could suggest that MUD primarily involves behavioral disinhibition rather than deficits in attention or future planning. These findings partially align with prior research, which has demonstrated a negative correlation between left pars opercularis thickness and the total, attention, and motor scores of the BIS-11 (Lim et al., 2021). In addition, performance levels on inhibitory control tasks have been correlated with gray matter intensity in the right pars opercularis region, which is associated with craving in healthy adults (Tabibnia et al., 2011). Furthermore, a correlation between right pars opercularis volume and internet addiction has been observed in females (Inhóf et al., 2019). A potential explanation for the observed discrepancies may be the differences in the research subjects.

### The role of trait impulsivity: as a mediator between right pars opercularis thickness and addiction severity

The chain mediation model, where the right pars opercularis thickness predicts addiction severity through MI and cue-induced craving, provides critical mechanistic clarity. Prior cross-sectional study struggled to disentangle whether impulsivity drives addiction or vice versa (Leshem & King, 2021). Our structural mediation evidence supports a neurodevelopmental cascade. First, METH-induced right pars opercularis thinning likely via dendritic spine loss (Nagy et al., 2024) impairs inhibitory control. Second, compromised inhibition elevates MI, which amplifies cue reactivity and craving (Bari & Robbins, 2013; Koob & Volkow, 2016). Third, this cycle culminates in escalated drug use and addiction severity. This model aligns with recent addiction vulnerability theories that emphasize prefrontal-striatal ‘braking system’ failure (Jahanshahi et al., 2015; Koob & Volkow, 2010). Importantly, our mediation analysis moves beyond correlation to indicate directionality: from structural integrity to behavior and subsequently to clinical outcomes. This finding has therapeutic significance, suggesting that neuromodulation targeting the right pars opercularis may break the cycle by enhancing inhibitory capacity.

### Limitations

This study presents several limitations. The first limitation pertains to the cross-sectional design of the study, which prevents the establishment of causal relationships between brain morphological measurements, trait impulsivity, drug craving, and METH addiction severity. Consequently, future research should incorporate longitudinal studies to effectively address these issues. The second limitation is that the study’s sample consisted solely of male participants, primarily recruited from a compulsory isolation and rehabilitation facility, and the institutional setting may have influenced self-reporting behaviors. This could potentially limit the generalizability of the results. Therefore, future research should involve multicenter studies to validate the findings of our study. In addition, while excluding ADHD enhances internal validity by reducing heterogeneity, it may also limit generalizability to the broader MUD population, suggesting that future studies ought to compare ADHD+/ADHD− MUD subgroups. Lastly, despite attempts to account for present smoking status and ever alcohol consumption as covariates in the analyses, significant disparities were observed in smoking status and alcohol consumption between the two groups. Therefore, it is imperative for future research to employ a robustly designed, multicenter, and longitudinal study to address these discrepancies.

## Conclusions

This study highlights key morphological abnormalities in the fusiform gyrus and pars opercularis, alongside disrupted small-world organization of SCNs, as neuropathological hallmarks of MUD. Furthermore, the mediating role of trait impulsivity and drug craving between cortical deficits and addiction severity emphasize impaired impulse control and reward processing as central mechanisms driving disease progression. Most notably, the right pars opercularis emerges as a promising neurobiological biomarker for maladaptive impulsivity in MUD, offering a potential target for therapeutic interventions. These findings refine the mechanistic understanding of MUD and support biomarker-informed strategies for diagnosis and treatment.

## Data Availability

The neuroimaging data are not publicly available due to their containing information that could compromise the privacy of the research participants.
